# Analysis of Expression of Vascular Endothelial Growth Factor A and Hypoxia Inducible Factor-1alpha in Patients Operated on Stage I Non-Small-Cell Lung Cancer

**DOI:** 10.1155/2014/810786

**Published:** 2014-02-10

**Authors:** Antonio Francisco Honguero Martínez, Antonio Arnau Obrer, Santiago Figueroa Almánzar, Pablo León Atance, Ricardo Guijarro Jorge

**Affiliations:** ^1^Thoracic Surgery, University General Hospital of Albacete, 37 Hermanos Falcó Street, 02006 Albacete, Spain; ^2^Thoracic Surgery, University General Hospital of Valencia and Surgery Department of the School of Medicine and Odontology, University of Valencia, 15 Blasco Ibáñez Avenue, 46010 Valencia, Spain; ^3^Thoracic Surgery, University General Hospital of Valencia, Tres Creus Avenue, 46014 Valencia, Spain

## Abstract

*Objectives*. Recent studies show that expression of hypoxia inducible factor-1alpha (HIF-1*α*) favours expression of vascular endothelial growth factor A (VEGF-A), and these biomarkers are linked to cellular proliferation, angiogenesis, and metastasis in different cancers. We analyze expression of HIF-1*α* and VEGF-A to clinicopathologic features and survival of patients operated on stage I non-small-cell lung cancer. *Methodology*. Prospective study of 52 patients operated on with stage I. Expression of VEGF-A and HIF-1*α* was performed through real-time quantitative polymerase chain reaction (qRT-PCR). *Results*. Mean age was 64.7 and 86.5% of patients were male. Stage IA represented 23.1% and stage IB 76.9%. Histology classification was 42.3% adenocarcinoma, 34.6% squamous cell carcinoma, and 23.1% others. Median survival was 81.0 months and 5-year survival 67.2%. There was correlation between HIF-1*α* and VEGF-A (*P* = 0.016). Patients with overexpression of HIF-1*α* had a tendency to better survival with marginal statistical significance (*P* = 0.062). Patients with overexpression of VEGF-A had worse survival, but not statistically significant (*P* = 0.133). *Conclusion*. The present study revealed that VEGF-A showed correlation with HIF-1*α*. HIF-1*α* had a tendency to protective effect with a *P* value close to statistical significance. VEGF-A showed a contrary effect but without statistical significance.

## 1. Introduction

Lung cancer is one of the most frequent neoplasms with a high global mortality [1, 2]. Prognosis is mainly determined by stage and TNM system, lastly revised in 2009 [3].

Stage I is the initial one and is every time more common due to the more extensive use of computed tomography scan on population. Surgery is the most efficient treatment on stage I. Nevertheless, although being an early stage and being treated by curative surgical resection, cancer related mortality is still high.

Oncology investigations in the last years are basically headed to study molecular biomarkers expression, cellular pathways, and interactions to better understand tumoral biology and develop effective treatment.

A lot of investigations on tumoral biomarkers in lung cancer early stages have been performed [4–6]. Angiogenesis is fundamental to favour tumoral growth [7, 8]. This process consists of formation of new vessels from preexisting ones to provide nutrients and physiological conditions needed to favour tumoral growth and development. Vascular endothelial growth factor A (VEGF-A) is the main angiogenic factor [9, 10] and is related to hypoxia inducible factor-1alpha (HIF-1*α*) owed to hypoxemic conditions surrounding tumoral growth [11, 12].

We performed a study about the expression of VEGF-A and HIF-1*α* and their prognosis on patients treated by surgery in stage I non-small-cell lung cancer.

## 2. Methodology 

### 2.1. Patients

From May 1, 2004 to December 31, 2007, a total of 66 patients took part in this study. All of them were operated on from non-small-cell lung cancer (NSCLC) and were classified into stage I after anatomopathologic study. Last 2009 TNM classification was used [3] and histology was done according to the World Health Organization classification [13]. Patients were excluded in the following cases: histology different to NSCLC, rejection to participate in the study, postoperative death (30 first days after intervention or until patient was discharged), tumor samples invalid to be processed, patients treated with induction therapy, or neoplasms in the 5 previous years other than basal cell carcinoma.

Preoperative work-up consisted of physical examination, blood analysis, electrocardiogram, chest X-ray film, and computed tomography (CT) scan of chest and upper abdomen, fiberbronchoscopy, spirometry, and electrocardiogram. Brain CT and bone scintigraphy were performed in case of clinical suspicion. Neither PET nor PET-CT scan was routinely performed because they were not available in that period. Preoperative mediastinal lymph nodes biopsy was performed when nodes' shortest diameter was superior to 1.0 cm to rule out N2 disease. After pulmonary resection, mediastinal lymph node dissection was done. In this study only patients without lymph node involvement after surgical resection were included.

Followup was done as follows: chest X-ray films two weeks after discharge to check for early postoperative complications, then, thorax and upper abdominal CT-scan every six months for the first two years, and afterwards one annual control in the next years. When tumour relapse was observed, histological confirmation was always attempted when feasible.

Patients signed informed consent and the study was approved by the local Bioethical Committee and by the Investigation Committee.

### 2.2. RNA Extraction and Reverse Transcription

After surgical resection, specimens of lung cancer and pulmonary parenchyma were collected in RNAlater (Invitrogen, USA) at –80°C. Total RNA was isolated from resected lung tissues using a RNA extraction reagent (TRI Reagent, Molecular Research Center, Sigma-Aldrich Inc., St. Louis, USA), following the manufacturer's instructions. Total RNA was digested with DNase at room temperature for 15 min. Five micrograms of digested RNA were reverse transcribed at 37°C for 120 min in a total reaction volume of 25 *μ*L, using the High-Capacity cDNA Reverse Transcription kit (Foster City, CA, USA).

The extracted RNA was analyzed by agarose gel electrophoresis. Only cases with preserved 28S, 18S indicating good RNA quality were included in this study. Obtained cDNA was used in quantitative real-time PCR reaction (qRT-PCR).

### 2.3. Detection of Gene Expression Using qRT-PCR

qRT-PCR was performed using master mix prepared according to the TaqMan Universal PCR Master Mix kit (Roche Molecular Systems Inc., Branchburg, NJ, USA). *β*-Actin was the housekeeping gene (Applied Biosystems, Assay ID: Hs99999903_m1. Amplicon size: 171 pb). VEGF-A and HIF-1*α* genes were also from Applied Biosystems:

 VEGF-A: assay ID: Hs00900054_m1, 77 pb amplicon;
 HIF-1*α*: assay ID: Hs00153153_m1, 76 pb amplicon.

qRT-PCR was carried out in a final volume of 50 *μ*L, with 0.05 *μ*g cDNA, 25 *μ*L TaqMan Universal PCR Master Mix (Roche Molecular Systems), and 1 *μ*M of each primer. Amplification was performed for 10 min at 95°C to activate AmpliTaq Gold Polymerase, then 40 rounds of 15 s at 95°C, and 1 min at 60°C for amplification and signal analysis. The device used to detect amplifications was ABI Prism 7000 Sequence Detection System from Applied Biosystems (USA). qRT-PCR data were automatically calculated with the data analysis module. Results were expressed as the ratio of expression in tumoral and lung tissues and normalized to the transcription level of the housekeeping gene. Validation of PCR efficiency was performed with a standard curve.

### 2.4. Statistical Analysis

Data were expressed as mean values ± standard deviation (SD) or median values and range. Association between categorical variables was performed using Chi-square or Fisher's exact test. For quantitative variables Student's *t*- or *U*-Mann-Whitney tests were used.

End of followup was December 31, 2010. The follow-up period was calculated from the date of surgical resection to death or last follow-up date for living patients. Alive and non-tumoral-cause dead patients were regarded as censored observations.

Overall survival curves were estimated using Kaplan-Meier method and compared by log-rank test. Cox proportional hazard regression model was used for multivariate analysis to evaluate the prognostic value of clinicopathological variables with respect to overall survival. All the applied tests were two-sided. The analysis was performed using a statistical software package (SPSS, Inc., version 12.0, Chicago, IL).

Statistical significance was defined as a value of *P* < 0.05.

## 3. Results

A total of 52 patients were included according to inclusion/exclusion criteria previously exposed. Male sex was predominant (86.5%) and adenocarcinoma subtype was the most frequent histology (Table 1). Stage IB was the most frequent (76.9%) and median follow-up time was 50 months (range: 1–92). Median survival time was 81.0 months and overall 5-year estimated survival was 67.2%.

**Figure 1:**
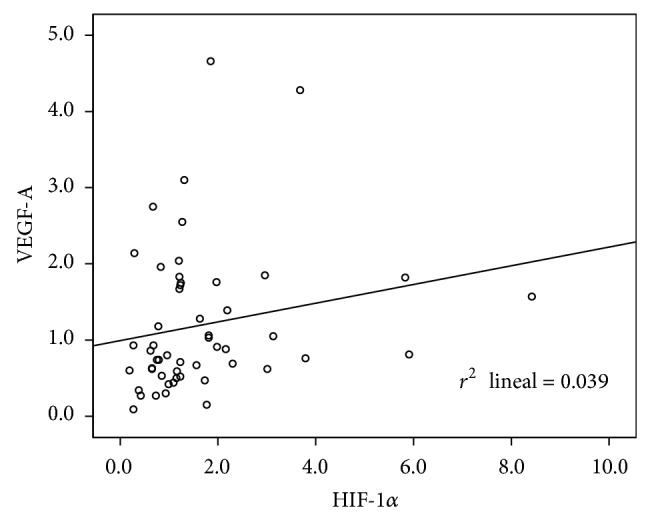
Scatter plot diagram between VEGF-A and HIF-1*α*. Determination coefficient (*r* ^2^) = 0.04. Correlation coefficient (*r*) = 0.33.

**Figure 2:**
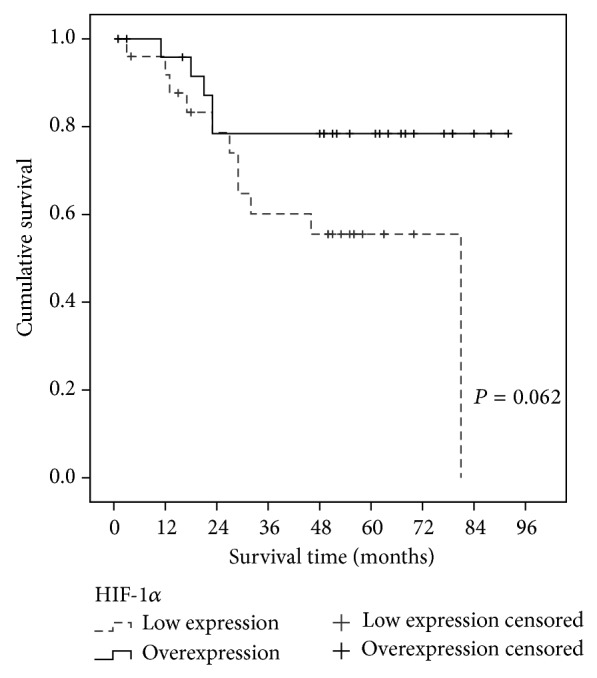
Survival curve according to expression of HIF-1*α* (log-rank test). Overexpression of HIF-1*α* when its level was superior to its median value (1.22).

**Figure 3:**
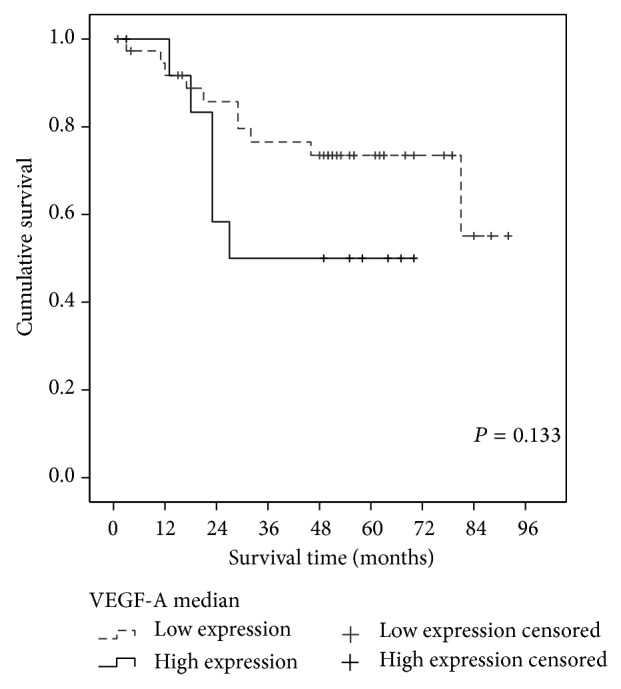
Survival curve according to the expression of VEGF-A (log-rank test). Overexpression of VEGF-A when its level was superior to percentile 75 (1.74).

Biomarkers VEGF-A and HIF-1*α* showed a nonnormal distribution. After checking quartile distribution and cancer-related deaths, cut-off points correspondent to 75 percentile (value = 1.74) and median (value = 1.22) values were chosen to consider the overexpression of VEGF-A and HIF-1*α*, respectively. Correlation between VEGF-A and HIF-1*α* as quantitative variables was statistically significant (*P* = 0.016) with Pearson's correlation coefficient of 0.33 (Figure 1). There was also statistical significance between HIF-1*α* and T descriptor (*P* = 0.001). This way, overexpression of HIF-1*α* in T2 tumors was higher than in T1 tumors (Table 2). Survival analysis revealed a tendency toward better prognosis in patients with HIF-1*α* overexpression (*P* = 0.062, Figure 2) and a tendency to a worse prognosis in patients with VEGF-A overexpression (Figure 3). At univariate analysis of Cox proportional hazard model on survival none of the variables reached statistical significance (Table 3). Nevertheless, HIF-1*α* showed the *P* value closest to statistical significance (*P* = 0.074) and an odds ratio value <1, reflecting its protective effect.

## 4. Discussion

In lung cancer, stage IA is one of the less frequent. It usually happens at radiology exploration (chest X-ray film, CT scan, etc.) performed for other reasons and a pulmonary solitary nodule is discovered. On the contrary, stage IB is the most frequent among patients operated on lung cancer. It encompasses tumors superior to 3 cm to up to 5 cm in diameter [3]. An early diagnosis is important in lung cancer due to its relevance on prognosis. However, lung cancer on early stage has a 5-year survival range between 73 and 57% [14, 15]. Currently, TNM system is the one used to classify disease extension and prognosis. It uses descriptors based on invasion or tumoral extension into anatomic or anatomopathologic elements, as a result of expression and evolution of molecular and cellular changes in cancer cells. In the last years, there is a proliferation of works involving biomolecular studies of cancer in general, and also lung cancer in particular, attending complicated pathways in cell proliferation, tumoral invasion, and metastasis [6, 16–20]. A solid tumor needs vascular vessels to provide them with nutrients to grow and a way to eliminate their metabolism-derived products.

This neovascularization arising from preexisting vessels is called neoangiogenesis [21]. When a tumor reaches more than 2-3 mm^3^ in size, a situation of cellular hypoxia occurs favouring expression of HIF-1*α*. This protein is translocated into the cellular nucleus and activates dozens of genes, such as VEGF-A [11, 22]. We observed a direct relation between VEGF-A and HIF-1*α* with statistical significance (*P* = 0.016), similar to that described by other authors [23, 24]. However, correlation coefficient (*r*) was low (0.33), as opposed to 0.67 observed by Kim et al. [25]. A possible explanation could be that overexpression of VEGF-A does not depend only on HIF-1*α*, but also other hypoxia-independent ways can produce overexpression of VEGF-A [11].

Related to tumoral size, there was statistical significance vinculated to HIF-1*α* (*P* = 0.001). Thus, overexpression of HIF-1*α* was higher on T2 than on T1 tumors. This finding could be linked to the fact that tumoral size increment favours a hypoxemic environment, so degradation of HIF-1*α* is decreased, and as a consequence HIF-1*α* is overexpressed [26].

At survival analysis, a tendency towards a worse prognosis of patients with overexpression of VEGF-A was observed, although without reaching statistical significance (0.133). In the scientific literature, most of the works and even meta-analysis found overexpression of VEGF-A as adverse prognosis factor on survival of NSCLC patients [27, 28]. Few works studied VEGF-A and HIF-1*α* together in lung cancer, and similar studies on early stage are scarce [23, 25]. These results may reflect at initial stage that VEGF-A does not take part so actively on tumoral development and metastasis, contrary to more advanced stages where its influence could be more evident. Nevertheless, a meta-analysis concluded that VEGF also presents worse prognosis in stage I. It could be probably due to more adenocarcinoma cases in the involved studies because prognosis was not found in squamous cases [27]. In our study, we did not find difference neither between VEGF-A nor HIF-1*α* overexpression and histology. With regard to HIF-1*α*, survival log-rank test showed a tendency to protective effect on patients with overexpression of HIF-1*α* with marginal statistical significance (0.062). Cox proportional hazards model showed an odds ratio inferior to zero (protective effect), although *P* > 0.05.

The role of HIF-1*α* in lung cancer has some controversy, and correlation between HIF-1*α* overexpression and adverse prognosis is not completely universal [11, 25, 29–31]. Thus, some authors found increment in survival and higher tumoral apoptosis rate among patients with HIF-1*α* overexpression [30, 31]. Moreover, relations between HIF-1*α* and proapoptotic proteins such as p53 or NIP3 have been described [32]. Also, HIF-1*α* polymorphisms and NSCLC susceptibility have been documented. This way, some variations in HIF-1*α* alleles can phenotypically manifest as more aggressive NSCLC [33]. In our series overexpression of HIF-1*α* was higher in T2 tumors, but no statistical significance was found at univariant analysis of prognostic factors on survival between T1 and T2, probably due to low number of patients with T1 tumors.

Molecular biology of cancer in general, and lung cancer in particular, is complex.

It is difficult to explain its development and evolution from expression of just a few genes. Knowledge and study of biomarkers are important to offer patients the best treatment according to their tumoral expression. In the future, it may be possible to offer every single patient a treatment in accordance with biomolecular profile expressed by his or her tumor [34]. In this way, studies in phase III have not yet been undergone administering bevacizumab (monoclonal antibody) in patients with overexpression of VEGF-A [35]. For this reason, it is necessary to carry out more studies on this subject to get a deeper knowledge and to establish a path steadier on prognosis and treatment of this disease.

In conclusion, correlation between HIF-1*α* and VEGF-A was observed in patients operated on stage I NSCLC. HIF-1*α* showed a tendency to protective effect on overall survival. VEGF-A overexpression showed an opposed effect (worse prognosis), although differences did not reach statistical significance.

## Figures and Tables

**Table 1:** Patients' clinicopathologic characteristics.

Age (mean ± s.d.)	64.7 years ± 10

Sex M : F (%)	45 (86.5%) : 7 (13.5%)

Histology	
Adenocarcinoma	22 (42.3%)
Epidermoid	18 (34.6%)
Others	12 (23.1%)

T1a	5 (9.6%)
T1b	7 (13.5%)
T2a	40 (76.9%)

Stage	
IA	12 (23.1%)
IB	40 (76.9%)

Alive patients	28 (53.8%)
Tumoral related death	16 (30.8%)
Nontumoral related death	8 (15.4%)

Tumoral size (mean ± s.d.)	3.03 cm ± 1.06

Tumoral relapse	
Yes	20 (38.5%)
No	32 (61.5%)

Smoking	
Never	4 (7.7%)
Former smoker	18 (34.6%)
Active smoker	30 (57.7%)

Follow-up time	
Median (range)	50 months (1–92)

Resection	
Sublobar	8 (15.4%)
Lobectomy	34 (65.4%)
Bilobectomy	3 (5.8%)
Pneumonectomy	7 (13.5%)

s.d.: standard deviation; M: male. F: female.

**Table 2:** Correlation of VEGF-A and HIF-1*α* with quantitative and qualitative variables.

	VEGF-A (*P*)	HIF-1*α* (*P*)
Quantitative variables		
Tumor size	0.134	0.175
Smoking (pack-year)	0.051	0.061
Preoperative haemoglobin	0.323	0.491
Age	0.298	0.347
VEGF-A	—	0.016∗
HIF-1*α*	0.016∗	—

Qualitative variables		
Sex	0.664	1.000
T1/T2	1.000	0.001∗
Histology	0.302	0.496
Recidive (yes/no)	0.211	0.910
State (alive/dead)	0.351	0.171

^*^Statistical significance (*P* < 0.05).

**Table 3:** Univariate analysis of prognostic factors on survival.

Variables	*P*	O.R. (C.I. 95%)
VEGF-A	0.145	2.162 (0.766–6.107)
HIF-1*α*	0.074	0.379 (0.131–1.100)
T1/T2	0.955	0.968 (0.312–3.003)
Histology	0.496	
Type of resection	0.180	

O.R.: odds ratio; C.I.: confidence interval.
